# Deletion of Gb3 Synthase in Mice Resulted in the Attenuation of Bone Formation via Decrease in Osteoblasts

**DOI:** 10.3390/ijms20184619

**Published:** 2019-09-18

**Authors:** Kazunori Hamamura, Kosuke Hamajima, Shoyoku Yo, Yoshitaka Mishima, Koichi Furukawa, Makoto Uchikawa, Yuji Kondo, Hironori Mori, Hisataka Kondo, Kenjiro Tanaka, Ken Miyazawa, Shigemi Goto, Akifumi Togari

**Affiliations:** 1 Department of Pharmacology, School of Dentistry, Aichi Gakuin University, Nagoya 464-8650, Japanag163d13@dpc.agu.ac.jp (S.Y.); lb_hidenori@yahoo.co.jp (Y.M.); hironori128@gmail.com (H.M.); KondoH@dpc.agu.ac.jp (H.K.); kenjiro1@dpc.agu.ac.jp (K.T.); togariaf@dpc.aichi-gakuin.ac.jp (A.T.); 2 Department of Orthodontics, School of Dentistry, Aichi Gakuin University, Nagoya 464-8650, Japan; miyaken@dpc.aichi-gakuin.ac.jp (K.M.); shig@dpc.agu.ac.jp (S.G.); 3 Department of Biomedical Sciences, Chubu University College of Life and Health Sciences, Kasugai, Aichi 487-8501, Japan; koichi@isc.chubu.ac.jp; 4 Japanese Red Cross Tokyo Blood Center, Tokyo 162-8639, Japan; m-uchikawa@ktks.bbc.jrc.or.jp; 5 Department of Biochemistry II, Nagoya University Graduate School of Medicine, Nagoya 464-8650, Japan; Yuji-Kondo@omrf.org

**Keywords:** glycosphingolipids, globoside (Gb4), osteoblasts, bone metabolism

## Abstract

Glycosphingolipids are known to play a role in developing and maintaining the integrity of various organs and tissues. Among glycosphingolipids, there are several reports on the involvement of gangliosides in bone metabolism. However, there have been no reports on the presence or absence of expression of globo-series glycosphingolipids in osteoblasts and osteoclasts, and the involvement of their glycosphingolipids in bone metabolism. In the present study, we investigated the presence or absence of globo-series glycosphingolipids such as Gb3 (globotriaosylceramide), Gb4 (globoside), and Gb5 (galactosyl globoside) in osteoblasts and osteoclasts, and the effects of genetic deletion of Gb3 synthase, which initiates the synthesis of globo-series glycosphingolipids on bone metabolism. Among Gb3, Gb4, and Gb5, only Gb4 was expressed in osteoblasts. However, these glycosphingolipids were not expressed in pre-osteoclasts and osteoclasts. Three-dimensional micro-computed tomography (3D-μCT) analysis revealed that femoral cancellous bone mass in Gb3 synthase-knockout (Gb3S KO) mice was lower than that in wild type (WT) mice. Calcein double labeling also revealed that bone formation in Gb3S KO mice was significantly lower than that in WT mice. Consistent with these results, the deficiency of Gb3 synthase in mice decreased the number of osteoblasts on the bone surface, and suppressed mRNA levels of osteogenic differentiation markers. On the other hand, osteoclast numbers on the bone surface and mRNA levels of osteoclast differentiation markers in Gb3S KO mice did not differ from WT mice. This study demonstrated that deletion of Gb3 synthase in mice decreases bone mass via attenuation of bone formation.

## 1. Introduction

Glycosphingolipids are considered to play an important role in the development and maintenance of various organs and tissues [1,2,3]. These have also been shown to be critical for protection against inflammation and degeneration [4]. Glycosphingolipids are divided into three main series, i.e., ganglioside-series, globo-series, and lacto/neolacto-series. Of these, gangliosides have been reported to be involved in bone metabolism [5,6,7,8,9]. For instance, it was reported that a-series ganglioside GD1a and GM1 promote osteoblastic differentiation of bone marrow mesenchymal stem cells (MSCs) and tendon stem cells [5,6,7,8]. Recently, we demonstrated that a lack of GD3 synthase, which is responsible for the generation of all b-series gangliosides, in mice results in the attenuation of bone loss with aging [9]. However, there have been no reports on the presence or absence of expression of globo-series glycosphingolipids in osteoblasts and osteoclasts, and the roles of these glycosphingolipids in bone metabolism.

**Figure 1:**
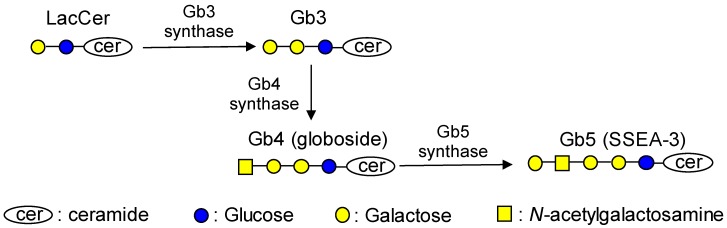
Synthetic pathway of globo-series glycosphingolipids.

Globo-series glycosphingolipids have a common core structure, Galα1,4-Galβ1,4-Glc-ceramide (globotriaosylceramide; Gb3), which is synthesized by α1,4-galactosyltransferase (Gb3 synthase *A4galt*) from lactosylceramide (LacCer) (Figure 1). Globo-series glycosphingolipids such as Gb3, Gb4 (globoside), and Gb5 (galactosyl globoside) have not only been used as markers for stem cells and tumors but are also considered to regulate immune system and maintain stemness [10,11,12,13]. Gb3 has been characterized as the P^k^ antigen on red blood cells [14] and is considered to be a tumor antigen of Burkitt lymphoma [10]. This glycosphingolipid has also been recognized as a receptor for verotoxins secreted by pathogenic *Escherichia coli* O157 [15]. Gb4 is a major component among glycosphingolipids in human erythrocytes [16] and is an essential structure for blood group P antigen [17]. Gb4 was also reported to be an endogenous ligand for Toll-like receptor 4-myeloid differentiation factor 2 (TLR4-MD-2) and attenuates lipopolysaccharide (LPS) toxicity [13]. Gb5, which is also named stage-specific embryonic antigen-3 (SSEA-3), is expressed at an early developmental stage and is used as a marker for stem cells [11].

In the present study, we used flow cytometry to examine the expression levels of globo-series glycosphingolipids (Gb3, Gb4, and Gb5) in MC3T3 E1 mouse osteoblast-like cells, SaM-1 human osteoblast cells, RAW264.7 mouse pre-osteoclasts, and primary cultured pre-osteoclasts derived from mouse bone marrow cells. To evaluate the involvement of globo-series glycosphingolipids in bone metabolism in vivo, femoral cancellous bone mass in wild type (WT) and Gb3 synthase-knockout (Gb3S KO) mice were analyzed using three-dimensional micro-computed tomography (3D-μCT). Furthermore, we conducted calcein double labeling to examine the roles of globo-series glycosphingolipids in bone formation. We employed bone histomorphometric analyses using hematoxylin and eosin (HE) and tartrate-resistant acid phosphatase (TRAP) staining. We also examined the expression levels of differentiation markers of osteoblasts and osteoclasts in long bones (femur and tibia) from WT and Gb3S KO mice using quantitative real-time PCR. 

We report here that deletion of Gb3 synthase in mice results in significant attenuation of bone formation, leading to decreased bone mass. This study is the first to report that Gb4 is expressed in osteoblasts, and also that globo-series glycosphingolipids are involved in bone metabolism. 

## 2. Results

### 2.1. Expression of Globo-Series Glycosphingolipids (Gb3, Gb4, and Gb5), and Gb3 and Gb4 Synthase Genes in Osteoblasts 

**Figure 2:**
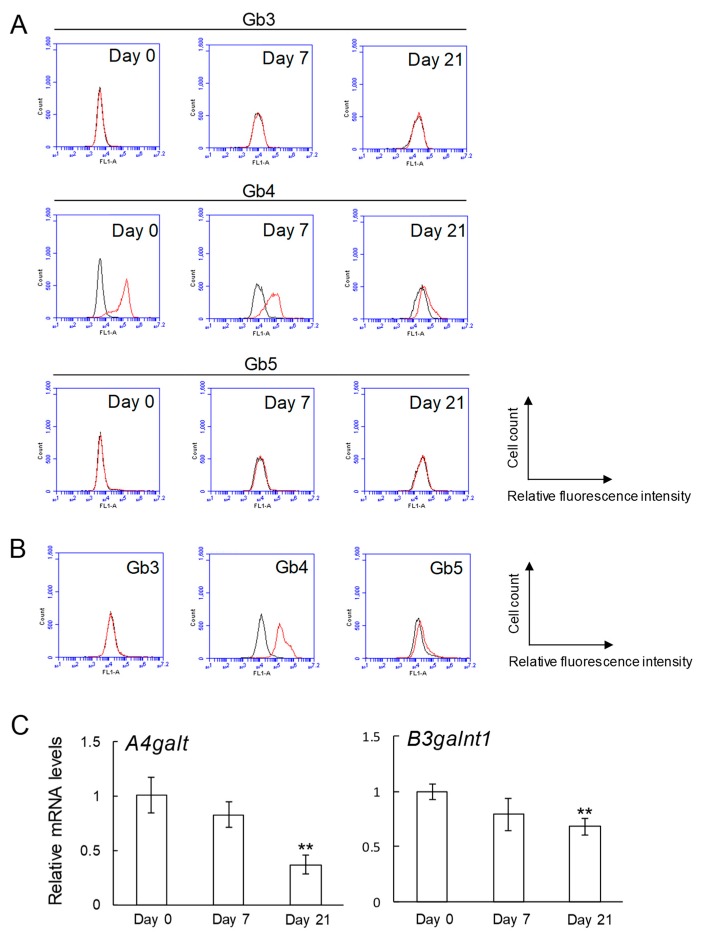
Expression of glycosphingolipids (Gb3, Gb4, and Gb5), and Gb3 and Gb4 synthase genes in osteoblasts. (**A**) Expression of globo-series glycosphingolipids (Gb3, Gb4, and Gb5) in MC3T3 E1 cells on days 0, 7, and 21 after induction of osteoblastogenesis by flow cytometric analysis. (**B**) Expression of globo-series glycosphingolipids (Gb3, Gb4, and Gb5) in SaM-1 cells by flow cytometric analysis. Red line: anti-globo-series glycosphingolipids (Gb3, Gb4, and Gb5) monoclonal antibodies (mAbs) (+), gray line: anti-globo-series glycosphingolipids mAbs (-). (**C**) mRNA expression of *A4galt* and *B3galnt1* in MC3T3 E1 cells on days 0, 7, and 21 after induction of osteoblastogenesis (*n* = 3). Data are expressed as mean ± S.D. The double asterisks indicate *p* < 0.01.

Expression levels of globo-series glycosphingolipids (Gb3, Gb4, and Gb5) in osteoblasts were analyzed by flow cytometry (Figure 2A,B). Both MC3T3 E1 mouse osteoblast-like cells and SaM-1 human osteoblast cells expressed Gb4 strongly, while Gb3 was below the limit of detection in both cells (Figure 2A,B). Gb5 in MC3T3 E1 cells was not detected and it was very weakly expressed in SaM-1. The expression of Gb4 in MC3T3 E1 cells was decreased after induction of osteoblastogenesis (Figure 2A). Consistent with this result, the expression levels of Gb3 (*A4galt*) and Gb4 (*B3galnt1*) synthase genes were downregulated by induction of osteoblastogenesis (Figure 2C).

### 2.2. No Expression of Globo-Series Glycosphingolipids (Gb3, Gb4, and Gb5), and Gb3 and Gb4 Synthase Genes in RAW264.7 Cells and Primary Cultured Pre-Osteoclasts

**Figure 3:**
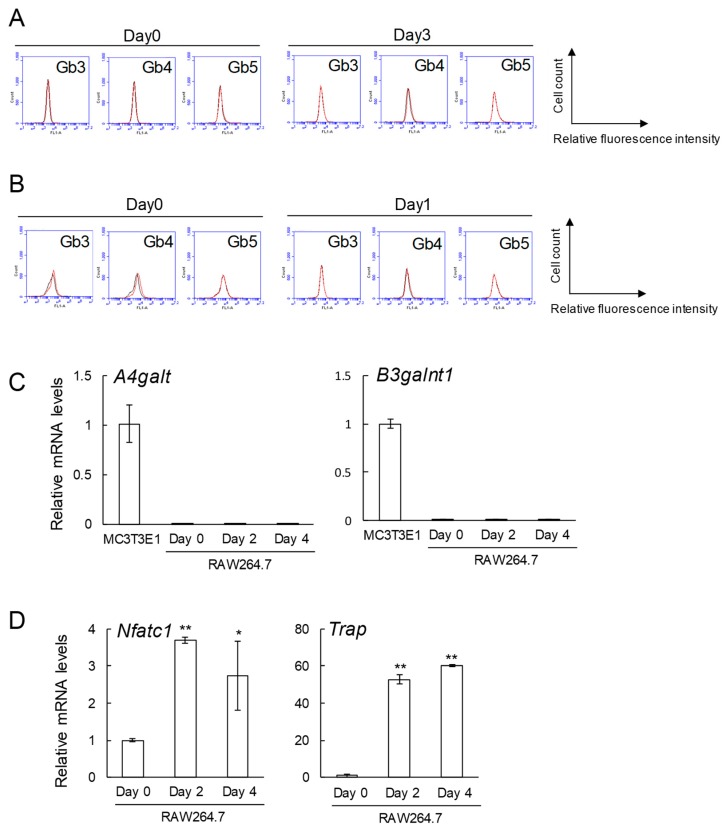
Expression of globo-series glycosphingolipids (Gb3, Gb4, and Gb5), Gb3 synthase gene (*A4galt*) and Gb4 synthase gene (*B3galnt1*) in RAW264.7 cells and primary cultured pre-osteoclasts in the presence or absence of RANKL. (**A**) Expression of globo-series glycosphingolipids (Gb3, Gb4, and Gb5) in RAW264.7 cells on days 0 and 3 after administration of RANKL by flow cytometric analysis. (**B**) Expression of globo-series glycosphingolipids (Gb3, Gb4, and Gb5) in primary cultured pre-osteoclasts on days 0 and 1 after administration of RANKL by flow cytometric analysis. Red line: anti-globo-series glycosphingolipids (Gb3, Gb4, and Gb5) monoclonal antibodies (mAbs) (+), gray line: anti-globo-series glycosphingolipids mAbs (-). (**C**) mRNA expression of *A4galt* and *B3galnt1* in MC3T3 E1 and RAW264.7 cells on days 0, 2, and 4 after administration of RANKL (*n* = 3). (**D**) mRNA expression of *Nfatc1* and *Trap* in RAW264.7 cells on days 0, 2, and 4 after administration of RANKL (*n* = 3). Data are expressed as mean ± S.D. The single and double asterisks indicate *p* < 0.05 and *p* < 0.01, respectively.

Gb3, Gb4, and Gb5 were not detected in RAW264.7 cells before (Day 0) and after (Day 3) induction to osteoclasts (Figure 3A). These glycosphingolipids were hardly expressed in primary cultured pre-osteoclasts derived from mouse bone marrow cells before (Day 0) and after (Day 1) induction to osteoclasts (Figure 3B). Consistent with the results of the flow cytometry, the expression levels of Gb3 (*A4galt*) and Gb4 (*B3galnt1*) synthase genes were at the limit of detection in RAW264.7 cells before (Day 0) and after induction (Day 2 and 4) to osteoclasts (Figure 3C). Of note, mRNA levels of osteoclast differentiation markers such as nuclear factor of activated T-cells, cytoplasmic 1 (*Nfatc1*) and tartrate-resistant acid phosphatase (*Trap*) were upregulated in the presence of the receptor activator of nuclear factor kappa-B ligand (RANKL) (Figure 3D). 

### 2.3. Decrease in Femoral Cancellous Bone Mass in Gb3 Synthase-Knockout (Gb3S KO) Mice 

**Figure 4:**
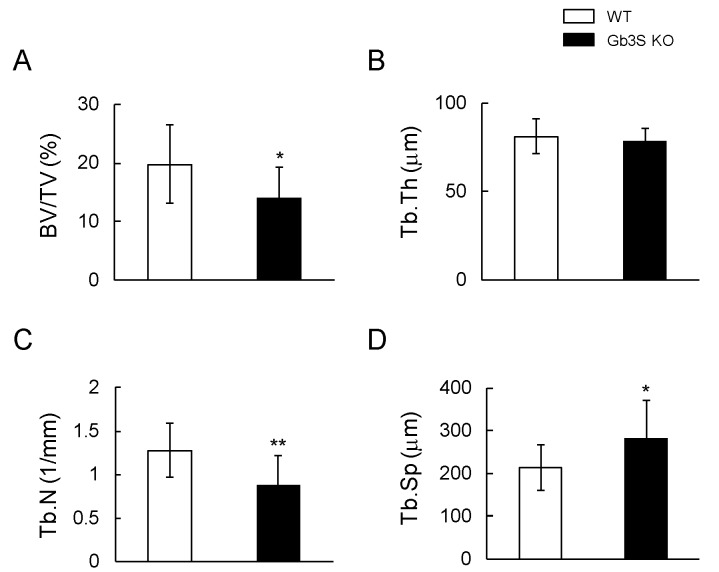
Attenuation of femoral cancellous bone mass in Gb3S KO mice. Analysis of bone densitometry of the distal region of the femur in WT and Gb3S KO mice by 3D-μCT. Fifteen week-old mice (male, *n* = 12 for WT, *n* = 11 for Gb3S KO). (**A**) Bone volume/total volume (BV/TV, %). (**B**) Trabecular thickness (Tb.Th, μm). (**C**) Trabecular number (Tb.N, 1/mm). (**D**) Trabecular separation (Tb.Sp, μm). Data are expressed as mean ± S.D. The single and double asterisks indicate *p* < 0.05 and *p* < 0.01, respectively.

Bone volume/total volume (BV/TV) and trabecular number (Tb.N) in Gb3S KO mice were significantly lower than those in WT mice (Figure 4A,C). Trabecular separation (Tb.Sp) was significantly higher than that in WT mice (Figure 4D). Trabecular thickness (Tb.Th) was not significantly different between WT and Gb3S KO mice (Figure 4B). The 3D-μCT analysis revealed that deletion of Gb3 synthase in mice results in the attenuation of bone mass. 

### 2.4. Decrease in Bone Formation Parameters by Gb3 Synthase Deficiency 

**Figure 5:**
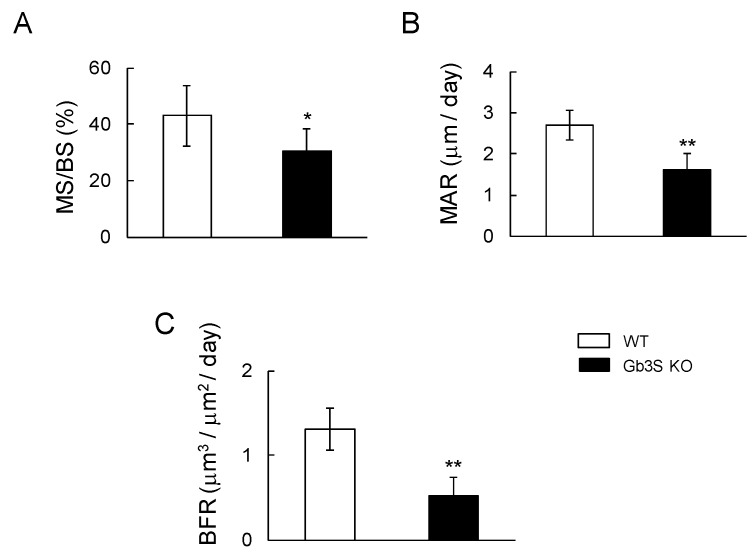
Attenuation of bone formation by Gb3 synthase deficiency in 15-week-old mice (male, *n* = 6 for WT, *n* = 6 for Gb3S KO). (**A**) Mineral surface/bone surface (MS/BS, %). (**B**) Mineral apposition rates (MAR, μm/day). (**C**) Bone formation rate (BFR, μm^3^/ μm^2^/day). Data are expressed as mean ± S.D. The single and double asterisks indicate *p* < 0.05 and *p* < 0.01, respectively.

To investigate whether Gb3 synthase regulates bone formation, we measured bone formation rate using calcein double labeling. The mineral surface/bone surface (MS/BS), mineral apposition rates (MAR), and bone formation rate (BFR) were significantly lower in Gb3S KO mice than in WT mice (Figure 5). 

### 2.5. Decrease in Osteoblast Number in Femoral Cancellous Bone and Osteogenic Differentiation Marker’s Gene Expression (Runt-Related Transcription Factor 2, Alkaline Phosphatase, and Osteocalcin) in Long Bones by Gb3 Synthase Deficiency 

**Figure 6:**
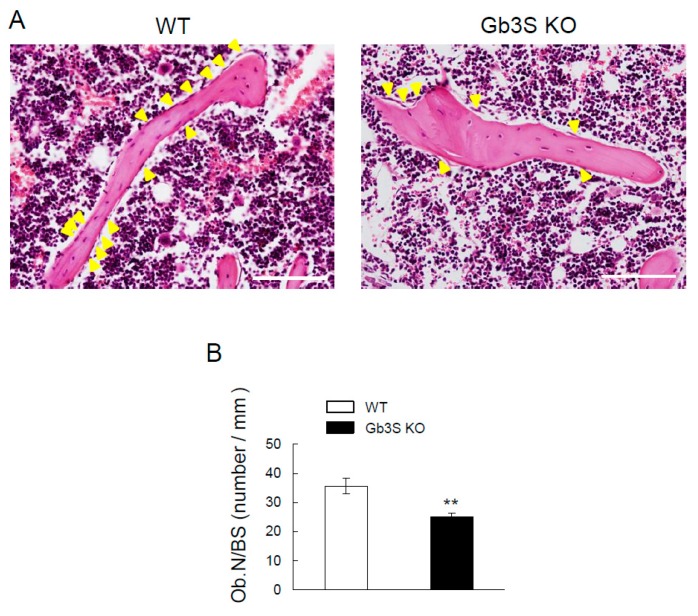
Decrease in osteoblast numbers by Gb3 synthase deficiency in 15-week-old mice (male, *n* = 10 for WT, *n* = 10 for Gb3S KO). (**A**) Images of hematoxylin and eosin (HE) staining of femoral cancellous bone. Results for WT mice (left) and Gb3S KO mice (right) are shown. The scale bars are 100 μm. Yellow arrow heads indicate osteoblasts. (**B**) Osteoblast numbers/bone surface (Ob.N/BS, number/mm). Data are expressed as mean ± S.D. The double asterisks indicate *p* < 0.01.

**Figure 7:**
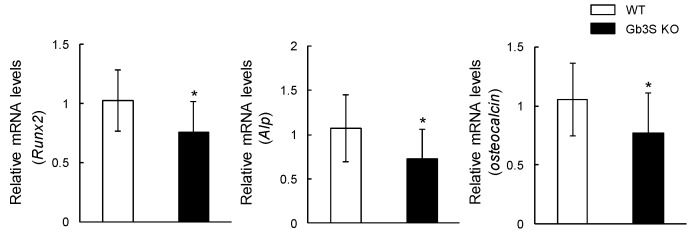
Suppression of gene expression of osteogenic differentiation markers (*Runx2*, *Alp*, and osteocalcin) by Gb3 synthase deficiency. RNA isolated from femur and tibia in 15-week-old mice (male, *n* = 12 for WT, *n* = 12 for Gb3S KO). Data are expressed as mean ± S.D. The single asterisk indicates *p* < 0.05.

Osteoblast numbers/bone surface (Ob.N/BS) was significantly lower in Gb3S KO mice than in WT mice (Figure 6A,B). Furthermore, mRNA levels of runt-related transcription factor 2 (*Runx2*), alkaline phosphatase (*Alp*), and osteocalcin were suppressed in Gb3S KO mice (Figure 7). 

### 2.6. No Differences in Osteoclast Number and Expression of Osteoclast Differentiation Marker’s Genes (Nuclear Factor of Activated T-Cells, Cytoplasmic 1 and Tartrate-Resistant Acid Phosphatase) between WT and Gb3S KO Mice 

**Figure 8:**
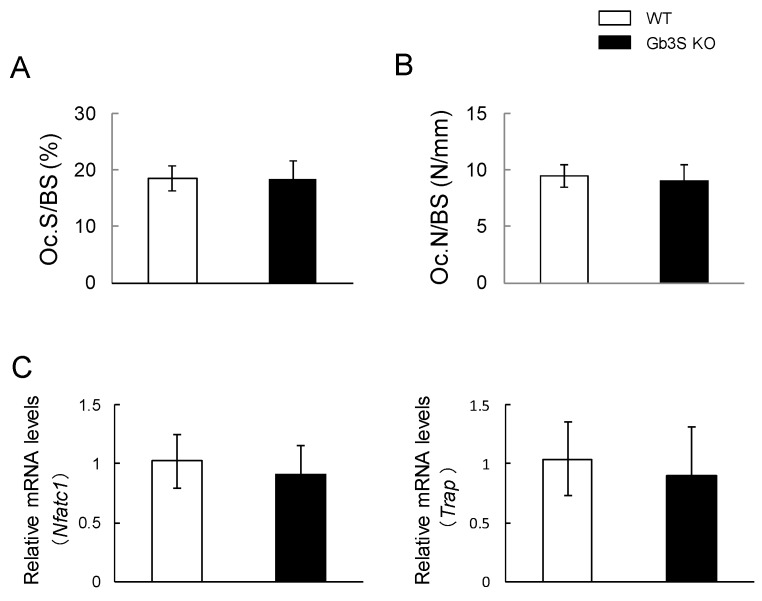
No effects of Gb3 synthase deficiency on bone resorption parameters and gene expression of osteoclast differentiation markers (*Nfatc1* and *Trap*). (**A**) Osteoclast surface/bone surface (Oc.S/BS, %) in 15-week-old mice (male, *n* = 9 for WT, *n* = 10 for Gb3S KO). (**B**) Osteoclast numbers/bone surface (Oc.N/BS, number/mm) in 15-week-old mice (male, *n* = 9 for WT, *n* = 10 for Gb3S KO). (**C**) mRNA expression of *Nfatc1* and *Trap*. RNA isolated from femur and tibia in 15-week-old mice (male, *n* = 12 for WT, *n* = 12 for Gb3S KO). Data are expressed as mean ± S.D.

Bone resorption parameters such as osteoclast surface/bone surface (Oc.S/BS) and osteoclast numbers/bone surface (Oc.N/BS) were almost equivalent in the WT and Gb3S KO mice (Figure 8A,B). Furthermore, no differences in nuclear factor of activated T-cells, cytoplasmic 1 (*Nfatc1*) and tartrate-resistant acid phosphatase (*Trap*) mRNA levels were found between WT and Gb3S KO mice (Figure 8C).

## 3. Discussion

In this study, we demonstrated that deficiency of Gb3 synthase in mice attenuates bone formation. Among Gb3, Gb4, and Gb5, only Gb4 was expressed in osteoblasts. However, none of these were expressed in pre-osteoclasts such as RAW264.7 and primary cultured pre-osteoclasts derived from mouse bone marrow cells. Furthermore, 3D-μCT, calcein double labeling, bone histomorphometric analyses, and expression analysis of osteogenic differentiation genes using Gb3S KO mice revealed that the deficiency of Gb3 synthase in mice decreased bone mass via attenuation of bone formation. 

It has been reported that among glycosphingolipids, gangliosides play an important role in the differentiation and proliferation of osteoblasts and osteoclasts. For instance, GD1a was reported to regulate differentiation from MSCs to osteoblasts via activation of epidermal growth factor receptor (EGFR) signaling [5,6]. GM1 also promotes differentiation from tendon stem cells to osteoblasts through attenuation of the phosphorylation of platelet-derived growth factor receptor-β (PDGFR-β) [8]. Furthermore, our recent study revealed that deficiency of GD3 synthase in mice results in the prevention of bone loss via the attenuation of bone resorption based on a decrease in osteoclast numbers [9].

This study revealed that genetic deletion of Gb3 synthase in mice decreased the number of osteoblasts and attenuated bone formation. Gb4 in osteoblasts may play a major role in this phenomenon, since osteoblasts did not express Gb3 and Gb5, but did express Gb4. This study also revealed that the expression of Gb4 in MC3T3 E1 cells was decreased after induction of osteoblastogenesis. We will need to examine which Gb4 is involved in the regulation of proliferation or differentiation in osteoblasts. To determine whether Gb4 in osteoblasts regulates the proliferation or differentiation, it is necessary to examine the effects of Gb4 treatment on osteoblasts derived from bone marrow or calvaria in Gb3S KO mice. 

Although there have been no reports that Gb4 regulates differentiation and proliferation of osteoblasts, it has been reported that this glycosphingolipid plays important roles in the regulation of cell signaling in various cells [18,19,20,21,22]. For instance, Gb4 promotes activation of extracellular signal-regulated kinases (ERK) signaling via association with EGFR [18] and is a potent inhibitor of protein kinase C (PKC) activity [19]. Gb4 also enhances the activity of transcription factors, activator protein-1 (AP-1) and cAMP response element binding protein (CREB) [20]. Furthermore, Gb4 upregulates Runx2 through TrkB-ERK signaling in dental epithelial cells [21]. Globo-series glycosphingolipids including Gb3 and Gb4, also increases the expression of P-glycoprotein through β-catenin signaling [22]. These signaling molecules such as ERK, PKC, AP-1, CREB, and β-catenin have been considered to be involved in proliferation and differentiation of osteoblasts [23,24,25,26]. 

**Figure 9:**
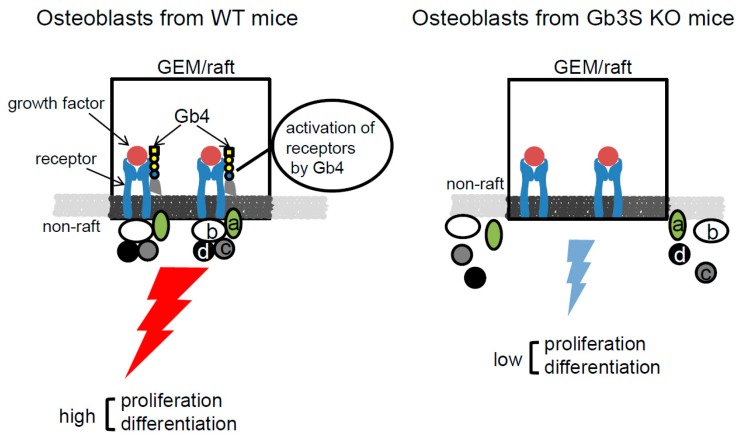
Schematic illustration of the proposed regulation of proliferation and/or differentiation of osteoblasts by Gb4.

Glycosphingolipids are enriched in glycolipid-enriched microdomain (GEM)/rafts on the plasma membrane of various cells [27], and enhance or attenuate the cell signals in the GEM/rafts [3,28,29,30,31]. Gb4 may also be localized in GEM/rafts, and interact with membrane molecules such as receptors, resulting in the formation of molecular complexes in the GEM/rafts, and promote the proliferation and/or differentiation of osteoblasts. Here, we propose the idea that Gb4 regulates the proliferation and/or differentiation of osteoblasts in GEM/rafts as shown in Figure 9.

The action mechanisms of globo-series glycosphingolipids in bone formation were not fully understood in this study. However, our observations provide the first evidence for the role of globo-series glycosphingolipids in bone formation. In summary, we have demonstrated that Gb4 is expressed in osteoblasts, and globo-series glycosphingolipids regulate bone mass through bone formation. 

## 4. Materials and Methods 

### 4.1. Mice

The generation of Gb3S KO mice was carried out as described previously [32]. WT and Gb3S KO mice were mated, and the resultant heterozygotes were mated, and genotypes of the offspring were screened as previously described [32]. The mice were housed under a 12 h light/dark cycle, and water and food were provided ad libitum. All protocols for animal experiments were approved by the Aichi Gakuin University Animal Research Committee (approval number AGUD348; 24 May 2016) (Nagoya, Japan), and were carried out in accordance with the National Institute of Health Guide for the Care and Use of Laboratory Animals (1966). 

### 4.2. Cell Culture

The MC3T3 E1 mouse osteoblast-like cells, SaM-1 human osteoblast cells [33], RAW264.7 mouse pre-osteoclast (monocyte/macrophage) cells, and mouse bone marrow cells isolated from long bones (femur and tibia) [34] were cultured in α-Minimum Essential Media with 10% fetal bovine serum and antibiotics (100 U/mL penicillin, 100 μg/mL streptomycin; Wako Pure Chemical Industries, Ltd., Osaka, Japan). Cells were maintained at 37 °C with 5% CO_2_ in a humidified incubator. 

### 4.3. Antibodies

Anti-Gb3 monoclonal antibody (mAb) c61 was generated in Dr. Furukawa’s laboratory [35]. Anti-Gb4 mAb HIRO34 was as described in [36]. Anti-Gb5 (SSEA-3) mAb and FITC-conjugated anti-mouse IgM were purchased from Affymetrix eBioscience (San Diego, CA, USA). FITC anti-human IgM and anti-rat IgM were purchased from BioLegend (San Diego, CA, USA). 

### 4.4. Induction to Mature Osteoblasts

The MC3T3 E1 cells were plated in 60 mm dishes. When cells were confluent, 50 μg/mL of ascorbic acid (Wako Pure Chemical Industries, Ltd.) and 5 mM β-glycerophosphate (Sigma-Aldrich, St. Louis, MO, USA) were added. The medium was changed every other day. After 7 and 21 days of culture, cells were used for flow cytometry or quantitative real-time PCR. 

### 4.5. In Vitro Osteoclast Induction

Mouse bone marrow cells were plated in 150 mm dishes and cultured with 10 ng/mL macrophage colony-stimulating factor (M-CSF; PeproTech, Inc., Rocky Hill, NJ, USA) for 3 days. The surface-attached cells were used as osteoclast precursors [34]. These precursors were cultured with 10 ng/mL M-CSF and 50 ng/mL RANKL (PeproTech, Inc.). After 24 h of treatment with RANKL, the cells were used for flow cytometry. RAW264.7 cells were plated in 150 mm dishes and cultured with 50 ng/mL RANKL. After 2, 3, and 4 days of treatment with RANKL, the cells were used for flow cytometry or quantitative real-time PCR.

### 4.6. Flow Cytometry

Cell surface expression of Gb3, Gb4, and Gb5 was analyzed by Accuri^TM^ C6 Flow Cytometer (BD Biosciences, San Jose, CA, USA) using anti-Gb3 (mouse IgM, c61), Gb4 (human IgM, HIRO34), and Gb5 (rat IgM, MC-631) mAbs. The cells were incubated with individual antibodies for 1 h on ice and then stained with FITC-conjugated anti-mouse, human, or rat IgM for 45 min on ice. Control cells for flow cytometry were prepared using the secondary antibody alone. 

### 4.7. Quantitative Real-Time PCR (qPCR)

Total RNA was extracted using a RNeasy Plus mini kit (Qiagen, Germantown, MD, USA). Reverse transcription was conducted with a high-capacity cDNA reverse transcription kit (Applied Biosystems, Carlsbad, CA, USA), and quantitative real-time PCR was performed using TaKaRa Thermal Cycler Dice Real Time System III with THUNDERBIRD SYBR qPCR mix kits (TOYOBO, Osaka, Japan). Total RNA was used for reverse transcription and the cycling conditions were 25°C for 10 min, 37°C for 120 min, and 85°C for 5 min. The PCR cycling conditions were 95°C for 10 min (pre-denaturation), 40 cycles at 95°C for 15 sec (denaturation), and 60°C for 1 min (extension) [9]. We evaluated the mRNA levels of *A4galt*, *B3galnt1*, *Runx2*, *Alp* (alkaline phosphatase), osteocalcin, *Nfatc1*, and *Trap* using primers listed in Table 1. *Gapdh* was used for an internal control. 

### 4.8. Three-Dimensional Micro-Computed Tomography (3D-μCT Analysis)

The distal region of the femur was scanned using 3D-μCT (CosmoScan R-mCT-GX-T1; RIGAKU, Tokyo, Japan), and the parameters of cancellous bone were analyzed by TRI/3D-BON (Ratoc, Tokyo, Japan) software as described previously [9]. In brief, the scanning was initiated at 0.5 mm above the distal femoral growth plate, and a total of 75 consecutive 20-μm-thick sections were analyzed. 

### 4.9. Measurement of Bone Formation Rate Using Calcein Double Labeling 

Calcein double labeling was performed to calculate mineral surface/bone surface (MS/BS), mineral apposition rates (MAR), and bone formation rate (BFR). We injected mice with calcein (8 μg/g; Sigma, St. Louis, MO, USA) intraperitoneally at 3 days and 1 day before euthanasia. The femurs were fixed with 4% paraformaldehyde and sectioned at 5 μm thickness as undecalcified sections. To measure MS/BS, MAR, and BFR, we used the metaphyseal cancellous bone in the femur to obtain the bone fraction in a rectangular area of 0.34 mm^2^ (0.5 mm × 0.67 mm), with its closest and furthest edges being 0.5 and 1.0 mm medial to the growth plate, respectively [37]. 

### 4.10. TRAP Staining 

TRAP staining was conducted as described previously [38]. In brief, the slides (femur samples) were incubated in sodium acetate buffer (0.1 M, pH 5.0) containing naphthol AS-MX phosphate, Fast Red Violet LB Salt, and MnCl_2_ in the presence of sodium tartrate at 37°C for 60 min for TRAP staining. 

### 4.11. Bone Histomorphometric Analyses 

Femur samples were fixed with 4% paraformaldehyde and decalcified in 10% ethylenediaminetetraacetic acid (EDTA) for three weeks, embedded in paraffin, and sectioned at 5 μm thickness. These slides were used for hematoxylin and eosin (HE) and TRAP staining. After staining with HE, osteoblast numbers on the bone surface (Ob.N/BS) were evaluated as described previously [9]. Osteoclasts were stained with TRAP, and osteoclast surface on the bone surface (Oc.S/BS) and osteoclast numbers on the bone surface (Oc.N/BS) were evaluated by scoring the TRAP-positive cells on the bone surface [9]. The parameters were measured within an area of 0.8 mm^2^ (1.0 mm × 0.8 mm), with the closest and furthest edges being 2.0 and 3.0 mm medial to the growth plate of the proximal ends of the femur, respectively [39]. 

### 4.12. Statistical Analysis

All data were expressed as mean ± S.D. Statistical significance was evaluated using a Student’s *t*-test at *p* < 0.05 and the single and double asterisks indicate *p* < 0.05 and *p* < 0.01, respectively.

## Figures and Tables

**Table 1:** Real-time PCR primers used in this study.

Target	Forward Primer	Backward Primer
*A4galt*	5′-CCTGTTCCCATCTGGAGGAG-3′	5′-CCCTTTCATCAGCACAACCA-3′
*B3galnt1*	5′-TCTTGACTGCCCTTCCCAAT-3′	5′-GGAGCGTGAAGCGAAAGTCT-3′
*Runx2*	5′-CCCAGCCACCTTTACCTACA-3′	5′-TATGGAGTGCTGCTGGTCTG-3′
*Alp*	5′-AACCCAGACACAAGCATTCC-3′	5′-GCCTTTGAGGTTTTTGGTCA-3′
*osteocalcin*	5′-CCGGGAGCAGTGTGAGCTTA-3′	5′-AGGCGGTCTTCAAGCCATACT-3′
*Nfatc1*	5′-GGTGCTGTCTGGCCATAACT-3′	5′-GCGGAAAGGTGGTATCTCAA-3′
*Trap*	5′-TCCTGGCTCAAAAAGCAGTT-3′	5′-ACATAGCCCACACCGTTCTC-3′
*Gapdh*	5′-TGCACCACCAACTGCTTAG-3′	5′-GGATGCAGGGATGATGTTC-3′
